# Monitoring phenylalanine concentrations in the follow‐up of phenylketonuria patients: An inventory of pre‐analytical and analytical variation

**DOI:** 10.1002/jmd2.12186

**Published:** 2020-11-22

**Authors:** Karlien L. M. Coene, Corrie Timmer, Susan M. I. Goorden, Amber E. ten Hoedt, Leo A. J. Kluijtmans, Mirian C. H. Janssen, Alexander J. M. Rennings, Hubertus C. M. T. Prinsen, Mirjam M. C. Wamelink, George J. G. Ruijter, Irene M. L. W. Körver‐Keularts, M. Rebecca Heiner‐Fokkema, Francjan J. van Spronsen, Carla E. Hollak, Frédéric M. Vaz, Annet M. Bosch, Marleen C. D. G. Huigen

**Affiliations:** ^1^ Translational Metabolic Laboratory, Department of Laboratory Medicine Radboud University Medical Centre Nijmegen The Netherlands; ^2^ Department Endocrinology and Metabolism Amsterdam UMC, University of Amsterdam Amsterdam The Netherlands; ^3^ Laboratory Genetic Metabolic Diseases, Department of Clinical Chemistry Amsterdam UMC, University of Amsterdam Amsterdam The Netherlands; ^4^ Department of Paediatrics, Division of Metabolic Disorders Amsterdam UMC, University of Amsterdam Amsterdam The Netherlands; ^5^ Department of Internal Medicine Radboud University Medical Centre Nijmegen The Netherlands; ^6^ Section Metabolic Diagnostics, Department of Genetics UMC Utrecht Utrecht The Netherlands; ^7^ Metabolic Laboratory, Department of Clinical Chemistry Amsterdam UMC, Vrije Universiteit Amsterdam Amsterdam The Netherlands; ^8^ Center for Lysosomal and Metabolic Diseases, Department of Clinical Genetics Erasmus MC Rotterdam The Netherlands; ^9^ Laboratory of Biochemical Genetics, Department of Clinical Genetics Maastricht University Medical Centre Maastricht The Netherlands; ^10^ Laboratory of Metabolic Diseases University of Groningen, University Medical Center Groningen Groningen The Netherlands; ^11^ Division of Metabolic Diseases Beatrix Children's Hospital, University Medical Centre Groningen Groningen The Netherlands

**Keywords:** bloodspot, DBS, hyperphenylalaninemia, laboratory variation, measurement, phenylalanine, phenylalanine, phenylketonuria, PKU

## Abstract

**Background:**

Reliable measurement of phenylalanine (Phe) is a prerequisite for adequate follow‐up of phenylketonuria (PKU) patients. However, previous studies have raised concerns on the intercomparability of plasma and dried blood spot (DBS) Phe results. In this study, we made an inventory of differences in (pre‐)analytical methodology used for Phe determination across Dutch laboratories, and compared DBS and plasma results.

**Methods:**

Through an online questionnaire, we assessed (pre‐)analytical Phe measurement procedures of seven Dutch metabolic laboratories. To investigate the difference between plasma and DBS Phe, participating laboratories received simultaneously collected plasma‐DBS sets from 23 PKU patients. In parallel, 40 sample sets of DBS spotted from either venous blood or capillary fingerprick were analyzed.

**Results:**

Our data show that there is no consistency on standard operating procedures for Phe measurement. The association of DBS to plasma Phe concentration exhibits substantial inter‐laboratory variation, ranging from a mean difference of −15.5% to +30.6% between plasma and DBS Phe concentrations. In addition, we found a mean difference of +5.8% in Phe concentration between capillary DBS and DBS prepared from venous blood.

**Conclusions:**

The results of our study point to substantial (pre‐)analytical variation in Phe measurements, implicating that bloodspot Phe results should be interpreted with caution, especially when no correction factor is applied. To minimize variation, we advocate pre‐analytical standardization and analytical harmonization of Phe measurements, including consensus on application of a correction factor to adjust DBS Phe to plasma concentrations.

**Abbreviations**
AAA: amino acid analyzer
Bias: average difference between test results obtained by two different methods
CI: confidence interval
CLSI: Clinical and Laboratory Standards Institute
CRM: Certified Reference Material: controls or standards used to validate analytical measurement methods
CV: coefficient of variation: a measure of the variability of the test results.
CV (%): SD/mean × 100
DBS: dried blood spot (DBSV = DBS of venous blood; DBSC = DBS of capillary blood)
EQA: external quality assessment
FIA‐MS/MS: flow‐injection analysis tandem mass spectrometry
FMOC: fluorenylmethyloxycarbonyl
IEC: ion exchange chromatography
LC‐MS/MS: liquid chromatography‐mass spectrometry
LoA: limits of agreement: mean bias ±1.96 × SD.
Phe: phenylalanine
PKU: phenylketonuria

## 1 INTRODUCTION

Phenylketonuria (PKU, OMIM 261600) is an autosomal recessive disorder of phenylalanine (Phe) metabolism caused by deficiency of phenylalanine hydroxylase (PAH), encoded by the *PAH* gene. If untreated, PKU can result in severe cognitive impairment and neurological abnormalities.^1^ These symptoms can largely be prevented by initiation of a Phe‐restricted diet shortly after birth. Maintaining adequate Phe concentrations is the strongest determinant for cognitive outcome in PKU patients.^1^, ^2^, ^3^ Therefore, regular laboratory monitoring of blood Phe concentrations is necessary to ensure adequate metabolic control. In the European guidelines, the advised range of blood Phe concentrations is 120 to 360 μmol/L for patients below 12 years of age and for patients during pregnancy, while for patients older than 12 years the advised range of blood Phe is 120 to 600 μmol/L. However, no preferred sample type, either dried blood spot (DBS) or plasma, is defined.^4^, ^5^

In the Netherlands, two main methods for measuring Phe concentrations are currently applied: analysis of the full amino acid spectrum in plasma by ion‐exchange chromatography with an amino acid analyzer or tandem mass‐spectrometry (MS/MS), and targeted measurement of Phe in DBS with MS/MS. For regular Phe monitoring, DBS is the preferred material, allowing patients to prepare their DBS at home and send it to the laboratory. Analysis of a full amino acid profile is considered as the gold standard method for Phe measurement. Recent studies have raised concerns that plasma and DBS methods do not render comparable Phe results.^6^, ^7^, ^8^, ^9^, ^10^

Besides different analysis techniques and origin of the blood sample (capillary or venous), there are multiple causes that can contribute to the variability of Phe measurements in DBS.^11^, ^12^ The European PKU guidelines acknowledged the high variation in Phe measurements, but did not address the possible differences between analysis‐ and sampling methods in much detail.^4^, ^5^ To provide further insight in the variation in Phe concentrations between the different analysis methods and between plasma and DBS, we performed an inter‐laboratory comparative study among seven metabolic laboratories in the Netherlands. We made an inventory of differences in pre‐analytical and analytical methodologies used for Phe determination, and compared differences in Phe results in DBS and plasma between and within the seven participating laboratories. In addition, we also studied differences in Phe concentrations between DBS prepared from capillary finger prick (DBSC) and from heparinized venous blood (DBSV).

## 2 METHODS

### 2.1 Inventory of Phe measurement methods in Dutch metabolic laboratories

All seven University Medical Center (UMC) laboratories for metabolic disorders in the Netherlands that provide care for PKU patients participated in this study: Radboudumc Nijmegen, Maastricht UMC+, Amsterdam UMC− location VUmc, Amsterdam UMC ‐ location AMC, Erasmus UMC Rotterdam, UMC Utrecht, and UMC Groningen. Through an online questionnaire, details were collected on Phe measuring methods, including information on standard operating procedures, analytical methodology, type of filter paper, type and size of puncher and whether a correction factor was used to convert DBS concentrations to approximate plasma concentrations.

### 2.2 Inclusion of patients

For the inter‐laboratory comparison, PKU patients who attended the adult outpatient clinic at Radboudumc Nijmegen for a regular follow‐up visit, including a blood draw for determination of Phe concentration, were eligible for inclusion in our study. All 23 patients (or their guardians) who were included approved of the use of their left‐over samples in an anonymized setting for method validation purposes, in agreement with institutional and national legislation and regulations for Good Clinical Practice. Inability to give informed consent was the sole exclusion criterion.

For the comparison of DBSC and DBSV Phe concentration, 41 PKU patients treated in the adult outpatient PKU clinic of the Amsterdam UMC, location AMC, were enrolled. Inability to give informed consent was the sole exclusion criterion. Samples were collected in two cohorts; in 2009/2010, 20 venous/capillary DBS sets were collected and in 2016, another 21 venous/capillary DBS sample sets were added to the study. In the last cohort, 1 capillary DBS sample was too small to measure and this set was excluded, resulting in a final set of 40 samples.

### 2.3 Preparation and distribution of plasma and DBS samples for analysis

From the blood samples of our outpatient‐clinic visitors, within 1 hour after sampling, 40 μL‐aliquots of heparinized whole blood were used to prepare DBS for all centers. All participating metabolic laboratories provided a sample of the specific filter paper used in their lab. All participating laboratories used their ISO 15189 accredited standard operating procedures for measurement of Phe in DBS, and for validation of filter paper used in this procedure. The filter papers in use in the different laboratories were not CLSI verified. Details on standard operating procedures and validation procedures are available on request from the different participating laboratories. Each laboratory received the DBS spotted on their own validated filter paper. After preparation of DBS, the heparin tube was centrifuged for 10 minutes at 2000*g* and plasma was aliquoted and stored at −20°C. DBS were sent to the participating laboratories on the day of preparation via regular mail. Plasma aliquots were shipped on dry ice to all participating laboratories in three separate batches. DBS and plasma samples were anonymized, and plasma batches were sent out in random order, so participating laboratories could not correlate DBS and plasma results. Every participating laboratory used their own validated analysis method. The results of the Phe measurements were sent to the organizing laboratory (Radboudumc) for data analysis. One laboratory (D) participated at a later point in the study and therefore only analyzed 12/23 DBS‐plasma combinations. Additionally, laboratories F and G both analyzed only 21 DBS‐plasma sets.

For the preparation of DBSC and DBSV, a capillary finger puncture was carried out to fill a blood spot (DBSC). At the same time (within 5 minutes) venous blood sampling using a heparin tube was performed. Within an hour of the blood draw, 40 μL of venous whole blood was spotted onto filter paper to prepare the DBSV. From both cards, Phe concentrations were analyzed by MS/MS in one laboratory, according to methods described by Rashed et al.^13^

### 2.4 Statistics

GraphPad Prism software v8.0 (GraphPad Software Inc.) was used for all statistical analyses. Pearson correlation coefficients (Pearson's *r*) were determined to assess the association between Phe concentrations and CV of blood spot and plasma measurements (across all laboratories).

To compare both methods within laboratories and to compare measurements from DBSC and DBSV, Bland‐Altman plots were generated. The relative difference in Phe value between the two methods or between the two different sample types is plotted on the Y‐axis. The relative differences are expressed as percentages of the mean Phe concentration. The X‐axis displays the mean Phe concentrations from every individual sample as generated by the two different methods or from the two different sample types. We have used the term bias here not in its strict statistical meaning, but to represent the average of all the differences, where we calculate difference as Plasma Phe minus DBSV Phe. The 95% limits of agreement describe the range for the true value. Additionally, methods/sample types were compared using Deming regression curves. For this analysis, we assumed that both methods have similar imprecision since they are assessed in the same units (orthogonal regression). If the confidence interval (CI) for the slope contains the value 1, it can be concluded that there is no proportional difference between the two methods. If the CI for the intercept contains the value 0, it can be concluded that there is no constant difference between the two methods. For the calculation of CVs and for the boxplots, all available data from the different laboratories was used. For within‐laboratory comparison of DBS and plasma Phe concentrations, only data was used when both plasma and DBS of an associated sample set were analyzed.

As the DBSC and DBSV data were collected in two separate batches, a Mann‐Whitney *U* test was performed to test whether the medians of these two batches were comparable. Because a nonsignificant *P*‐value (.234) was found, the null hypothesis of equal medians was accepted and therefore the data was combined.

## 3 RESULTS

### 3.1 Variability in Phe analysis methods among metabolic laboratories in the Netherlands

All seven Dutch metabolic laboratories consented to participate in this study, filled out the digital questionnaire, and sent in filter paper for preparation of DBS. The results of the questionnaire showed that each individual laboratory developed its own specific standard operating procedure. There was no consistency on analytical method, type, and size of puncher or the use of a correction factor to convert Phe concentrations in DBS to approximate plasma concentrations.

Table 1 gives an overview of the different methods currently used in clinical practice in the Netherlands for measuring Phe concentrations in plasma and DBS.

**TABLE 1:** Overview of methods used for determination of Phe concentration in plasma and DBS among seven Dutch metabolic laboratories

Lab	Method Phe plasma	Derivatization DBS method	Calibration method	Filter paper source	Punch diameter (mm)	Estimated volume of blood in punch (μL)	Correction factor^a^	Inter‐assay CV DBS method (%)
A	LC‐MS/MS	Butylation	Aqueous calibrator	Whatman CF12 collection paper	1.5	1.27	1.50	9
B	Amino‐acid analyzer	FMOC	Aqueous calibrator	Whatman 903 protein saver card	5.5	8.1	None	10
C	Amino‐acid analyzer	None	Aqueous calibrator	Local supplier, 182 g/m^2^ filter paper	4.76	7.45	None	8
D	Amino‐acid analyzer	Butylation	Calibration through internal standard	Local supplier, 180 g/m^2^ filter paper	6.35	10	1.19	8.7
E	Amino‐acid analyzer	Butylation	Calibration through internal standard	Whatman 903 protein saver card	6.35	10	1.11	7.4
F	LC‐MS/MS	None	Aqueous calibrator	Local supplier, details unknown	6.0	11	None	6
G	Amino‐acid analyzer	None	Aqueous calibrator	Sartorius TFN Grade, 179 g/m^2^ filter paper	3.2	3.1	1.28	9.6

Abbreviations: DBS, dried blood spot; FMOC, fluorenylmethyloxycarbonyl; LC‐MS, liquid chromatography‐mass spectrometry.

^a^ The correction factor indicates whether an individual laboratory adjusts the Phe concentration measured in DBS with a specific factor to better approximate the corresponding plasma concentration, “none” means that specific laboratory did not apply a correction factor. Of note, all laboratories used an LC‐MS/MS method for DBS Phe measurement and 100% methanol for DBS extraction.

### 3.2 Inter‐laboratory comparison of Phe measurements in plasma and blood spot

**FIGURE 1:**
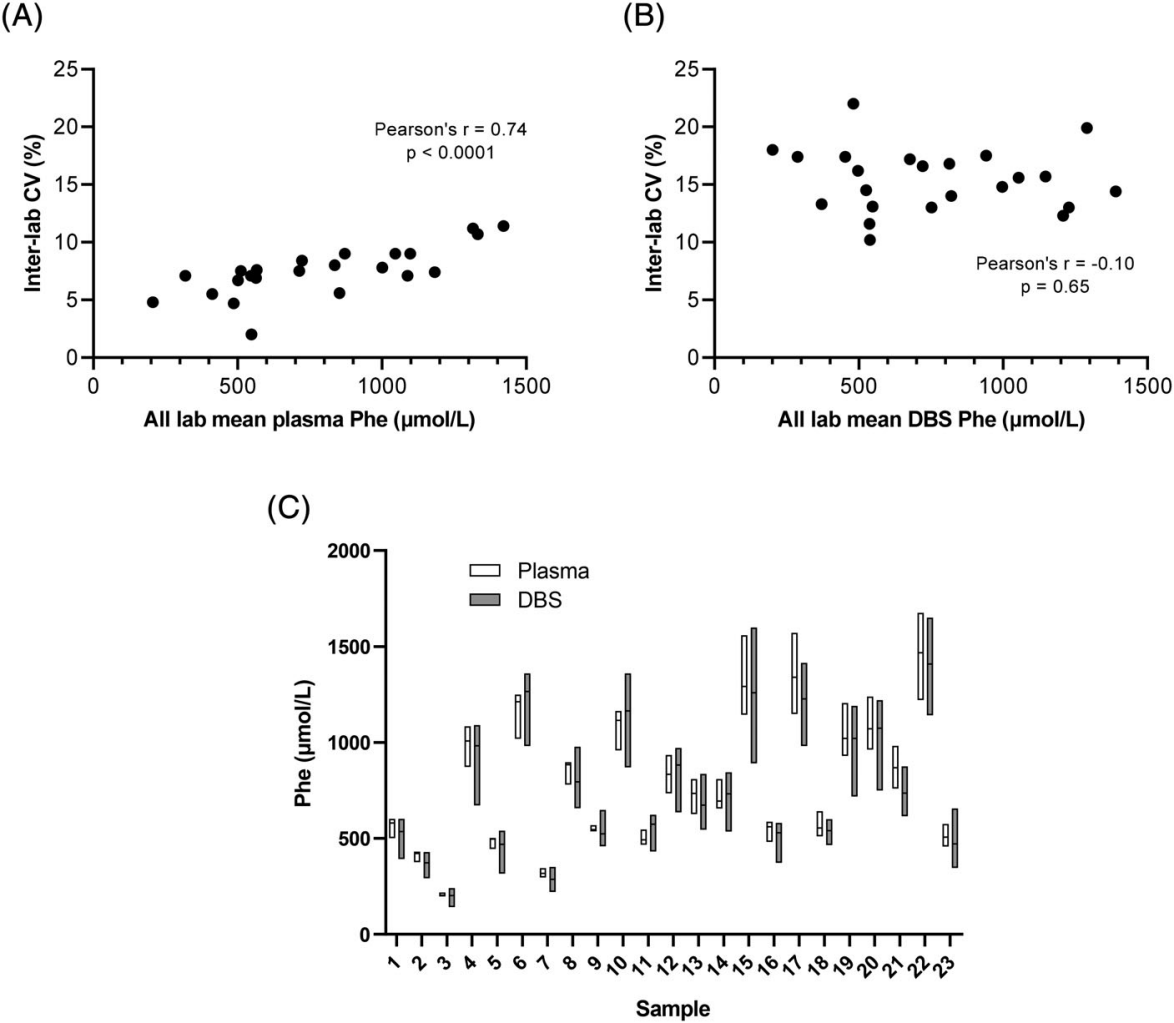
Inter‐laboratory coefficient of variation (CV) of plasma, A, and DBSV, B, Phe concentrations as measured across the seven participating laboratories; dried bloodspot (DBS) data shown in this figure involve a bloodspot prepared from venous blood (DBSV). Pearson correlation coefficient (Pearson's *r*) is indicated in the figure. C, Overview of plasma and DBSV Phe concentrations as measured in the different sample sets (1‐23) by the seven participating laboratories. The box extends from the minimum to maximum μmol/l Phe concentration; the horizontal line in the box indicates the median Phe concentration (numerical data shown in Table S1)

Twenty‐three PKU patients (9 males and 14 females, median age 28 years, range 16‐59 years) agreed to participate in this study. We first analyzed the inter‐laboratory coefficient of variation (CV) for Phe concentrations in plasma. In our study, the mean inter‐laboratory CV was found to range from 2.0% to 11.4%. The inter‐laboratory CV increased with higher plasma Phe concentrations (Figure 1A). In Figure 1B, the inter‐laboratory CV is plotted against concentration for the DBS measurements. It is evident that the inter‐laboratory variation for Phe in DBS is higher than for plasma, ranging from 11.6% to 22.0%, without increasing with higher Phe concentrations. This finding in DBS is comparable with the 20.2% inter‐laboratory variation described by Moat et al^9^ in a study in 16 laboratories in the United Kingdom. Figure 1C and Table S1 show an overview of all results for the 23 sets of plasma and associated DBS sample.

### 3.3 Inter‐laboratory comparison of association of Phe concentration between bloodspot and plasma

**FIGURE 2:**
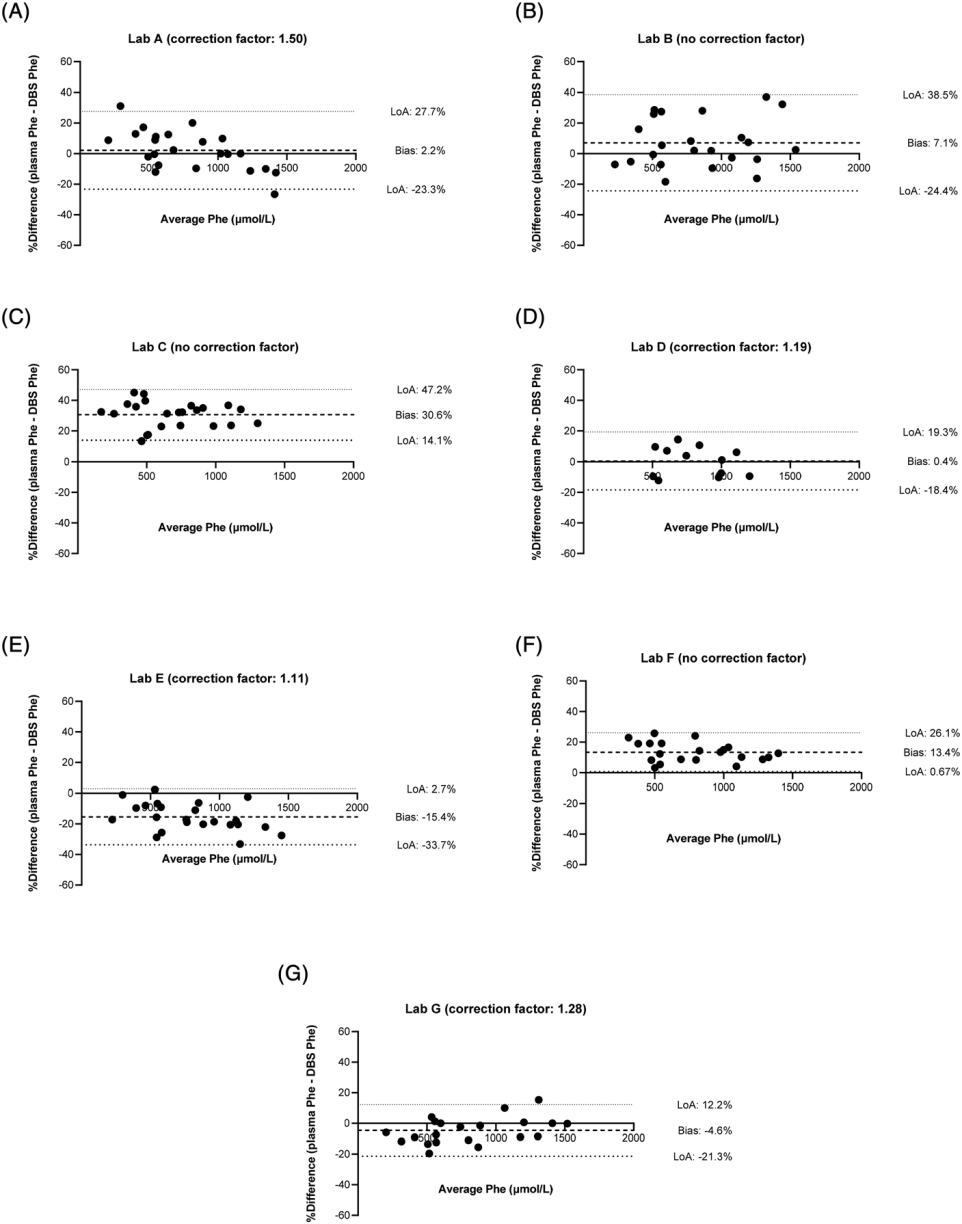
Bland‐Altman (BA) analysis of plasma and DBSV Phe concentrations, plotted for every individual participating laboratory, A‐G, dried bloodspot (DBS) data shown in this figure involve a bloodspot prepared from venous blood (DBSV). Biases (average difference in %) and limits of agreement (LoA) are indicated by dashed lines. The relative difference in Phe value between the two methods is plotted on the Y‐axis. The relative differences are expressed as percentages of the mean Phe concentration. The X‐axis displays the mean Phe concentrations from every individual sample as generated by the two different methods. The bias represents the average of all the differences, while the 95% limits of agreement describe the range for the true value

Next, we analyzed associations between DBS and plasma Phe concentrations for the individual laboratories. The difference between the two measurements is shown in Bland‐Altman plots in Figure 2, in which the average percentage difference is indicated by the term bias. The average difference between DBS and plasma Phe concentration for an individual laboratory ranged from −15.4% to +30.6%. For the four laboratories who corrected DBS Phe concentrations to approximate plasma concentrations, average differences ranged from −15.4% to +2.2%, while for laboratories that did not apply a correction factor, average difference ranged from +7.1% to +30.6%. In Figure S1, also the Deming regressions curves of the association between plasma and DBS Phe are depicted for individual laboratories. From these results, it becomes apparent that substantial inter‐laboratory differences exist in the comparability of DBS Phe to plasma Phe concentrations.

### 3.4 Comparison of capillary blood spots with blood spots prepared from venous sampling

**FIGURE 3:**
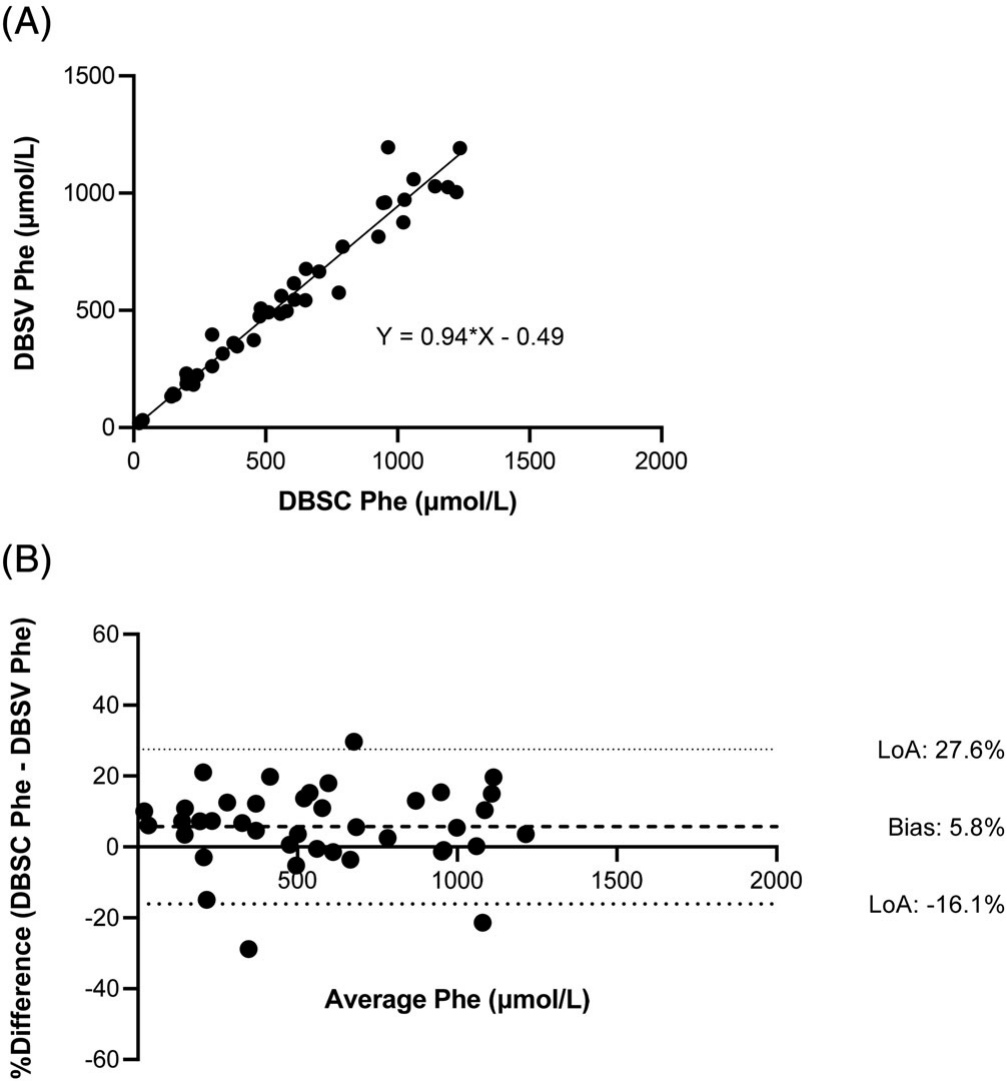
Phe concentrations in dried bloodspot (DBS) taken venously (DBSV) vs capillary (DBSC), plotted as Deming regression curve, A, and Bland‐Altman plot, B. The relative difference in Phe value between the two different sample types is plotted on the Y‐axis. The relative differences are expressed as percentages of the mean Phe concentration. The X‐axis displays the mean Phe concentrations from every individual sample as generated from the two different sample types. The bias here represents the average of all the differences, while the 95% limits of agreement describe the range for the true value

For practical reasons, we chose to prepare DBS from venous whole blood for the inter‐laboratory comparison study (DBSV). However, in daily clinical practice, PKU patients prepare blood spots at home from capillary blood obtained by finger prick (DBSC). We wanted to gain further insight in the differences between DBSC and DBSV Phe concentrations. Therefore, 40 DBSC and DBSV sample sets were prepared from 30 adult PKU patients (11 males and 19 females), with a median age of 29 years (range 18‐47 years). Comparison of DBSC vs DBSV Phe concentrations indicated that on average, DBSC render 5.8% higher Phe concentrations than DBSV. However, the 95% limits of agreement were wide, ranging from −16.1% to 27.6% (Figure 3).

## 4 CONCLUSION AND DISCUSSION

Reliable measurement of Phe concentration is crucial to monitor and adjust individual treatment for PKU patients. Previous studies have raised concern on the comparability of Phe concentrations measured in plasma vs DBS,^6^, ^7^, ^8^ the latter most frequently used because of patient convenience. Our findings in a large collaborative study among all metabolic laboratories in the Netherlands confirm these concerns and emphasize the high variability in (pre‐)analytical procedures between individual laboratories for Phe measurement in DBS. The average difference between plasma Phe and DBSV Phe concentrations reported by the seven different laboratories ranged from −15.4% to +30.6% (plasma Phe 15.4% lower to 30.6% higher than DBSV Phe). A reported Phe concentration from DBS at the upper limits of the critical treatment‐range of either 360 or 600 μmoL/L, respectively, could therefore represent a plasma Phe concentration ranging from 305 to 470 μmoL/L or 508 to 784 μmoL/L, respectively. This illustrates how the laboratory variation reported here could potentially give rise to differences in clinical advise between centers.

The difference between plasma and DBS Phe was more pronounced for laboratories that did not apply a correction factor to relate DBS Phe concentrations to plasma levels, resulting in DBS Phe concentrations which were 7.1% to 30.6% lower compared to Phe concentrations in the corresponding plasma, which is comparable to findings from previous studies.^6^, ^7^, ^8^ However, it must be noted that the analytical methods used for Phe measurement in these latter studies were based on flow‐injection analysis (FIA‐MS), while the methods that we have compared in this study all included liquid chromatography separation methods preceding MS analysis (LC‐MS). The four laboratories in our study that applied a correction factor to adjust DBS Phe concentrations to plasma concentrations had an average difference ranging from −15.4% to +2.2% (plasma Phe 15.4% lower to 2.2% higher than DBS Phe). This suggests that the applied correction to approximate the plasma Phe concentration indeed better reflects the measured plasma Phe concentration. Of note, the finding that laboratory E reported a 15.4% lower Phe concentration in plasma than in DBSV, even after a correction factor was applied to the latter, was remarkable. When DBS Phe external quality assessment (EQA) results during the timeperiod of the study of this laboratory were checked retrospectively, indeed it was apparent that also in EQA, laboratory E showed a positive bias for DBS results. An explanation was then found in the volume of the punch estimated by laboratory E, which appeared to be too low. In any case, for an individual measurement, the difference between plasma Phe and DBS can be substantial, also because the within‐laboratory analytical variation of an individual measurement will contribute to this difference.

Additionally, we show that the type of sample to prepare DBS (capillary blood from a finger prick vs heparinized venous blood) likely contributes to the variation in Phe measurements. In the one center where this was studied, DBSV on average yielded 5.8% lower Phe results than DBSC, while Mo et al^14^ showed opposite results in healthy controls, and Wagner et al^15^ state there is little knowledge on the impact of the matrix composition (DBSV vs DBSC) on Phe determination. We took the DBSC directly from the finger, therefore the blood volume applied for DBSC was not standardized, but we chose this approach as it best reflects the situation in clinical practice. In a recent study from Vliet et al,^10^ no significant difference between DBSV and DBSC was reported. However, in this study Passing‐Bablok regression analysis was used, which is less sensitive to outliers and also does not consider measurement error in both variables. Also, the age distribution between the two study populations for this comparison was different, as our study population did not include children, which may have contributed to an age‐related effect. Future studies should further investigate the relation between DBSC and DBSV results.

Apart from the analytical method and the material of choice, additionally sample volume, type of filter paper, extraction efficiency of Phe from DBS, punch location in DBS (central vs peripheral) and overall sample quality are likely to further contribute to pre‐analytical variation for DBS Phe results.^9^, ^11^, ^12^, ^16^ In this study, the type/batch of filter paper for DBS differed between laboratories, but each laboratory made use of filter paper that was validated according to ISO 15189 standards for their own use, even though extraction efficiency was not known to all laboratories. The different filter papers had comparable proportions in the absorbance of blood, as the punch diameter appeared to correlate to the estimated volume in the punch, based on the information shown in Table 1. The participating laboratories also used their own uncertified Phe calibration material with potentially suboptimal purity, which could further add up to the observed variation. An additional source of variation that is underestimated in our study is the actual preparation of the DBS in the patient's home. In our study, the preparation was performed in a controlled setting, with a single technician preparing the DBSV samples and with guidance from a researcher in the preparation of DBS from capillary finger prick. When DBSC is prepared by (parents of) patients at home, which is the usual situation, one can assume that the variation in DBS quality is much higher than in the current study.^9^, ^11^, ^16^ In addition, there were only samples from adults included in this study, while especially for children or neonates more difficulties in adequate filling of the DBS can be expected, as well as effects of higher hematocrit. Because of these additional sources of variation, the differences in Phe concentrations measured from DBS compared to plasma are likely to be even higher in daily clinical practice than reported in this study.

In conclusion, the findings presented in the current study emphasize the practical issues with Phe measurements for follow‐up of PKU patients. We show a high variability in methods between different laboratories and differences in an individual laboratory between Phe measurements in plasma or DBS. Phe measurements which are fundamental to adequate clinical management of PKU patients are clearly laboratory and sample type dependent. This is an undesirable situation, as it may result in different treatment advice between, and likely even within individual centers, even when the same clinical guidelines are adhered to.

The Phe target ranges as posed in all PKU guidelines, including the European guidelines,^4^, ^5^ are partly based on studies without clearly defined methodology, including specification on sample types and correction factor used for Phe measurements. With our current findings, we aim to increase awareness of the substantial (pre‐)analytical variation in Phe measurements for PKU follow‐up, and point out that absolute Phe target ranges from literature should be applied with caution. To reduce the issues of variability in Phe concentrations, we advocate pre‐analytical standardization, and analytical harmonization of methodologies between different laboratories where possible. For the specific situation in the Netherlands, ideally an equal analytical procedure should be implemented in the different laboratories for DBS Phe measurement. As a practical starting point toward this end, harmonization should at least involve the use of standardized paper and puncher to prepare DBS and standardized calibration methodology. Also, it should be required for every laboratory to apply a correction factor, determined using a standardized procedure, to correlate DBS to plasma Phe results, and to participate in EQA such as the ERNDIM DBS Phe programme. In light of transparency, future publications on Phe concentrations in PKU management should specify the analytical method and sample type used for Phe measurement. In case of DBS measurement, also the correction factor applied to adjust DBS Phe concentrations to approximate plasma Phe concentrations should be clearly stated. Finally, we strongly advise to provide regular training for patients and carers on DBS blood collection techniques to ensure optimal DBS quality. By taking these steps, our final goal is to ensure optimal, personalized care for every PKU patient.

## CONFLICT OF INTERESTS

C. E. H. has been involved in premarketing studies with Sanofi‐Genzyme, Protalix, and Idorsia; A. M. B. has been a member of the advisory board of Biomarin and received speaker fee from Nutricia; F. J. van S. is/has been a member/chair of the following advisory boards: Biomarin, Agios, Applied Pharma Research, Arla Food Int. Eurocept, Lucane, Nestle‐Codexis Alliance, Nutricia, Orphan Europe, Origin, Rivium Medical BV, Sobi, Vivet, NKUV, NPKUA, and ESPKU, has received research grants from BioMarin, Codexis, ESPKU, NPKUA, NPKUV, Nutricia, Sobi, and Tyrosinemia Foundation, is/has been consultant to Applied Pharma Research, BioMarin, Nutricia, Orphan Europe, Pluvia Biotech, has given lectures for which his institute received financial compensation: BioMarin, MendeliKABS, and Nutricia. Apart from this, the University Medical Center of Groningen is involved in a research project—so far without financial compensation—to invent a home monitoring device. K. L. M. C., C. T., S. M. I. G., A. E. ten H., L. A. J. K., M. C. H. J., A. J. M. R., H. C. M. T. P., M. M. C. W., G. J. G. R., I. M. L. W. K.‐K., M. R. H.‐F., F. M. V. and M. C. D. G. H. declared no conflict of interests.

## AUTHOR CONTRIBUTIONS

**Karlien L. M. Coene**, **Corrie Timmer**, **Susan M. I. Goorden**, **Annet M. Bosch**, and **Marleen C. D. G. Huigen**: Made substantial contributions to conception and design, analysis and interpretation of data. **Karlien L. M. Coene**, **Corrie Timmer**, and **Susan M. I. Goorden**: Wrote the first draft of the manuscript. All authors have participated in acquisition of data, drafting the manuscript or revising it critically for important intellectual content. All authors have given their final agreement to the submission after inspection.

## INFORMED CONSENT

For the inter‐laboratory comparison, patient approval of the use of their left‐over samples in an anonymized setting for method validation purposes was given, in agreement with institutional and national legislation and regulations for Good Clinical Practice. For the comparison of DBSC and DBSV Phe concentration, ethical approval was obtained from the AMC ethics committee, and all patients provided signed informed consent. All procedures followed were in accordance with the ethical standards of the responsible committee on human experimentation (institutional and national) and with the Helsinki Declaration of 1975, as revised in 2000.

## Supporting information


**FIGURE S1** Deming regressions curves of plasma and DBSV Phe association for individual laboratories (S1A‐S1G). The equation of the Deming regression curve is shown in the plot. (S1H) 95% Confidence Intervals (CI) of the slope and Y‐intercept for the different labs. If the CI for the slope contains the value 1, it can be concluded that there is no proportional difference between the two methods. If the CI for the intercept contains the value 0, it can be concluded that there is no constant difference between the two methods.

**TABLE S1** Overview of results for different plasma‐ DBSV sample sets (visual representation is shown in Figure 1C).
