# Supplementary material for: Monitoring phenylalanine concentrations in the follow‐up of phenylketonuria patients: An inventory of pre‐analytical and analytical variation
Source: JIMD Rep. 2020 Nov 22;58(1):70–9. doi: 10.1002/jmd2.12186 (PMC7932865; doi:10.1002/jmd2.12186)
Supplement: Supplementary file 1 — FIGURE S1 Deming regressions curves of plasma and DBSV Phe association for individual laboratories (S1A‐S1G). The equation of the Deming regression curve is shown in the plot. (S1H) 95% Confidence Intervals (CI) of the slope and Y‐intercept for the different labs. If the CI for the slope contains the value 1, it can be concluded that there is no proportional difference between the two methods. If the CI for the intercept contains the value 0, it can be concluded that there is no constant difference between the two methods. [file JMD2-58-70-s001.pptx]

## Slide 1
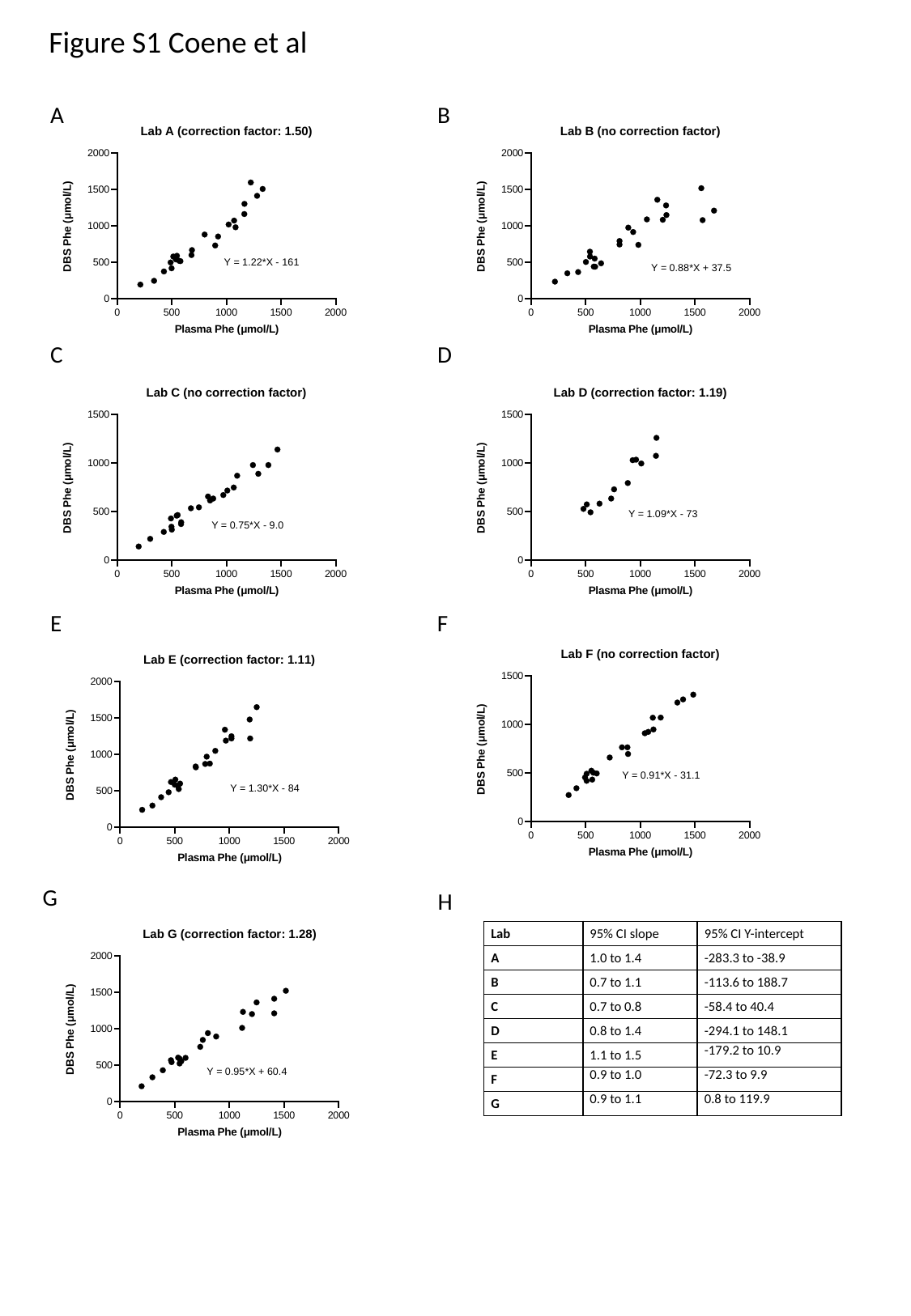

Figure S1 Coene et al
B
A
D
C
F
E
G
H
| Lab | 95% CI slope | 95% CI Y-intercept |
| --- | --- | --- |
| A | 1.0 to 1.4 | -283.3 to -38.9 |
| B | 0.7 to 1.1 | -113.6 to 188.7 |
| C | 0.7 to 0.8 | -58.4 to 40.4 |
| D | 0.8 to 1.4 | -294.1 to 148.1 |
| E | 1.1 to 1.5 | -179.2 to 10.9 |
| F | 0.9 to 1.0 | -72.3 to 9.9 |
| G | 0.9 to 1.1 | 0.8 to 119.9 |
