# Supplementary material for: Monitoring phenylalanine concentrations in the follow‐up of phenylketonuria patients: An inventory of pre‐analytical and analytical variation
Source: JIMD Rep. 2020 Nov 22;58(1):70–9. doi: 10.1002/jmd2.12186 (PMC7932865; doi:10.1002/jmd2.12186)
Supplement: Supplementary file 2 — TABLE S1 Overview of results for different plasma‐ DBSV sample sets (visual representation is shown in Figure 1C). [file JMD2-58-70-s002.docx]

*Table S1. Overview of results for different plasma- DBSV sample sets (visual representation is shown in Figure 1C).*

|  | **Mean plasma Phe (μmol/l)** | **Median plasma Phe (μmol/l)** | **Range plasma Phe (μmol/l)** | **Inter-lab CV plasma (%)** | **Mean**  **DBSV Phe (μmol/l)** | **Median DBSV Phe** | **Range DBSV Phe (μmol/l)** | **Inter-lab CV DBS (%)** |
| --- | --- | --- | --- | --- | --- | --- | --- | --- |
| **Set 1** | 564 | 581 | 501-602 | 6.9 | 525 | 535 | 392-602 | 14.5 |
| **Set 2** | 412 | 422 | 376-432 | 5.5 | 371 | 372 | 292-429 | 13.3 |
| **Set 3** | 206 | 203 | 197-219 | 4.8 | 201 | 202 | 142-241 | 18.0 |
| **Set 4** | 1001 | 1007 | 872-1084 | 7.8 | 941 | 982 | 672-1090 | 17.5 |
| **Set 5** | 486 | 497 | 445-503 | 4.7 | 453 | 469 | 316-540 | 17.4 |
| **Set 6** | 1183 | 1212 | 1019-1249 | 7.4 | 1208 | 1266 | 981-1360 | 12.3 |
| **Set 7** | 319 | 318 | 296-345 | 7.1 | 288 | 287 | 221-351 | 17.4 |
| **Set 8** | 853 | 883 | 779-897 | 5.6 | 820 | 795 | 657-978 | 14.0 |
| **Set 9** | 548 | 545 | 537-569 | 2.0 | 537 | 524 | 459-648 | 11.6 |
| **Set 10** | 1089 | 1115 | 959-1164 | 7.1 | 1147 | 1164 | 870-1360 | 15.7 |
| **Set 11** | 501 | 493 | 466-547 | 6.7 | 548 | 574 | 431-623 | 13.1 |
| **Set 12** | 836 | 833 | 734-935 | 8.0 | 814 | 882 | 635-971 | 16.8 |
| **Set 13** | 723 | 734 | 627-810 | 8.4 | 677 | 673 | 545-836 | 17.2 |
| **Set 14** | 714 | 694 | 655-810 | 7.5 | 721 | 732 | 536-845 | 16.6 |
| **Set 15** | 1315 | 1292 | 1144-1558 | 11.2 | 1290 | 1259 | 891-1598 | 19.9 |
| **Set 16** | 546 | 561 | 481-587 | 7.1 | 497 | 529 | 373-580 | 16.2 |
| **Set 17** | 1332 | 1339 | 1148-1571 | 10.7 | 1228 | 1227 | 981-1414 | 13.0 |
| **Set 18** | 566 | 554 | 510-642 | 7.6 | 539 | 540 | 465-601 | 10.2 |
| **Set 19** | 1046 | 1021 | 930-1206 | 9.0 | 997 | 1021 | 718-1190 | 14.8 |
| **Set 20** | 1098 | 1071 | 962-1239 | 9.0 | 1054 | 1074 | 749-1220 | 15.6 |
| **Set 21** | 871 | 869 | 760-983 | 9.0 | 752 | 736 | 615-875 | 13.0 |
| **Set 22** | 1421 | 1467 | 1221-1675 | 11.4 | 1390 | 1409 | 1141-1650 | 14.4 |
| **Set 23** | 511 | 506 | 458-575 | 7.5 | 481 | 471 | 346-655 | 22.0 |
